# Is Longitudinal Division in Rod-Shaped Bacteria a Matter of Swapping Axis?

**DOI:** 10.3389/fmicb.2018.00822

**Published:** 2018-05-08

**Authors:** Tanneke den Blaauwen

**Affiliations:** Bacterial Cell Biology and Physiology, Swammerdam Institute for Life Sciences, University of Amsterdam, Amsterdam, Netherlands

**Keywords:** division, FtsZ, morphology, MreB, peptidoglycan, symbionts

## Abstract

The morphology of bacterial species shows a wealth of variation from star-shaped to spherical and rod- to spiral-shaped, to mention a few. Their mode of growth and division is also very diverse and flexible ranging from polar growth and lateral surface increase to midcell expansion and from perpendicular to longitudinal asymmetric division. Gammaproteobacterial rod-shaped species such as *Escherchia coli* divide perpendicularly and grow in length, whereas the genetically very similar rod-shaped symbiotic *Thiosymbion* divide longitudinally, and some species even divide asynchronously while growing in width. The ovococcal *Streptococcus pneumoniae* also lengthens and divides perpendicularly, yet it is genetically very different from *E. coli*. Are these differences as dramatic as is suggested by visual inspection, or can they all be achieved by subtle variation in the regulation of the same protein complexes that synthesize the cell envelope? Most bacteria rely on the cytoskeletal polymer FtsZ to organize cell division, but only a subset of species use the actin homolog MreB for length growth, although some of them are morphologically not that different. Poles are usually negative determinant for cell division. Curved cell poles can be inert or active with respect to peptidoglycan synthesis, can localize chemotaxis and other sensing proteins or other bacterial equipment, such as pili, depending on the species. But what is actually the definition of a pole? This review discusses the possible common denominators for growth and division of distinct and similar bacterial species.

## Introduction

Bacteria can grow with a wealth of variations (Kysela et al., 2016) of which only the morphogenesis of the sphere, the ovococ, the rod-shape, and the crescent-shaped species have been thoroughly investigated in the form of *Staphyloccocus aureus* (Monteiro et al., 2015; Wheeler et al., 2015)*, Streptococcus pneumoniae* (Morlot et al., 2003; Land and Winkler, 2011; Fleurie et al., 2014; Tsui et al., 2014; Bajaj et al., 2016), *Escherichia coli* (Egan et al., 2015; Blaauwen et al., 2017), *Bacillus subtilis* (Adams and Errington, 2009), and *Caulobacter crescentus* (Yakhnina and Gitai, 2013; Collier, 2016; Woldemeskel and Goley, 2017), respectively. More recently, other rod-shaped bacteria such as *Myxococcus xanthus* that grow by elongation at lateral sides (Lotte Søgaard-Andersen personal communication) and rods that grow by extension of their poles [*Mycobacterium tuberculosis* (Singh et al., 2013), *Corynebacterium glutamicum* (Letek et al., 2008), and *Agrobacterium tumefaciens* (Cameron et al., 2015)], from midcell (Rhizobiales; Brown et al., 2012), or as branched filaments (*Streptomyces*; Jakimowicz and van Wezel, 2012; Claessen et al., 2014), are receiving more attention (Eswara and Ramamurthi, 2017). This development is applaudable as it is discovered that our laboratory pets are not the standard for morphological organization and regulation we thought them to be.

A better understanding of the available variation in the organization of bacterial growth (see for a review Yang et al., 2016) will enable the discrimination of key factors that determine survival of bacteria. Likely, if new antibiotics are to be discovered they should preferably interfere with multiple of these key factors to postpone resistance as long as possible. In fact, that is what many of the naturally occurring antibiotics such as beta-lactams and fluoroquinolones do (Hooper, 2001; Cho et al., 2014) and why they have been very successful, until we have started to apply them in quantities that were not meant to be used. Consequently, a dramatic increase in antibiotic resistance developed to a point that some bacterial infections have become untreatable (http://www.who.int/mediacentre/news/releases/2017/bacteria-antibiotics-needed). In addition, detailed molecular knowledge of specific species could be of use for the development of new small spectrum antibiotics, to diminish destruction of the composition of the gut microbiota (Gao et al., 2017).

**Figure 1:**
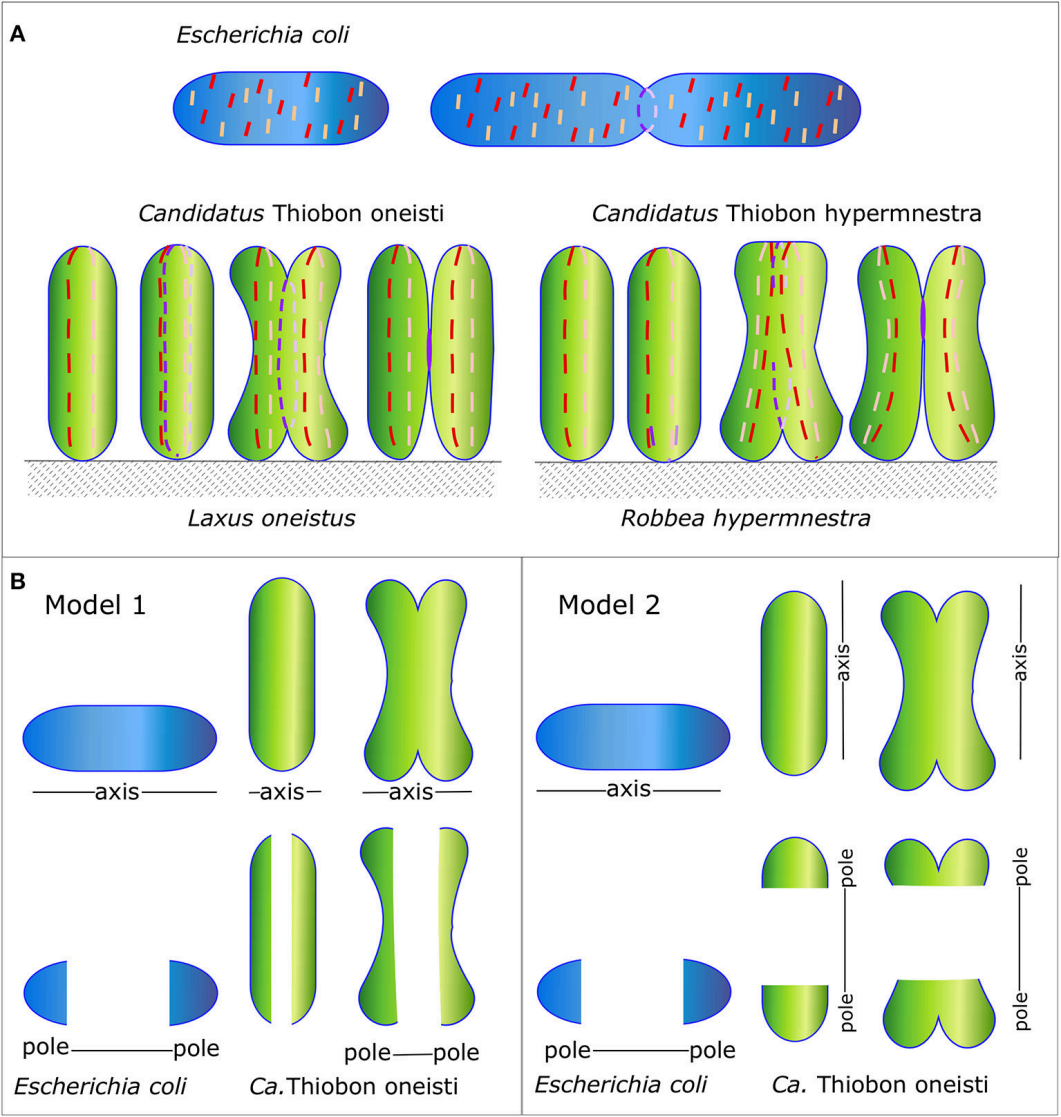
Schematic representation of the Morphology of *E. coli* and *Ca*. T. oneisti and *Ca*. T. hypermnestrae and their MreB and FtsZ localization patterns. **(A)** Orientation of the division plane in the gammaproteobacteria *E. coli* (in blue) and *Ca*.T. oneisti and *Ca*. T. hypermnestae (in green). The very simplified localization of MreB and FtsZ are shown as a dashed red and purple line, respectively. **(B)** Two possible models. *Ca*. T. oneisti can be seen as a short very fat *E. coli* (model 1) or as a cell that has swapped its major axes in comparison to *E. coli* (model 2).

Even among phylogenetically related species, considerable difference in morphology can be observed despite a very high genomic similarity. For instance, the gammaproteobacteria *Escherichia coli, Candidatus* Thiosymbion oneisti, and *Candidatus* Thiosymbion hypermnestrae, share most of the genes known to be involved in morphogenesis, whereas their division modes are very different. The Thiosymbion live in marine sediment attached via one of their cell poles to the cuticle of their nematode host, *Laxus oneistus* and *Robbea hypermnestra*, respectively. *E. coli* is a rod-shaped bacterium that divides perpendicularly to produce two identical daughter cells, whereas the rod-shaped symbionts have approximately the same size as *E. coli* but divide longitudinally (Leisch et al., 2012, 2016; Figure 1A). Presumably, they have evolved to divide longitudinally to ensure the daughter cells have an anchor point on the nematode surface. These bacteria can be seen as very fat and short *E. coli* that still divide perpendicularly (Figure 1B model 1) or as thin and long bacteria that divide longitudinally (Figure 1B model 2). This review will try to discriminate between the two models.

## Peptidoglycan determines shape

**Figure 2:**
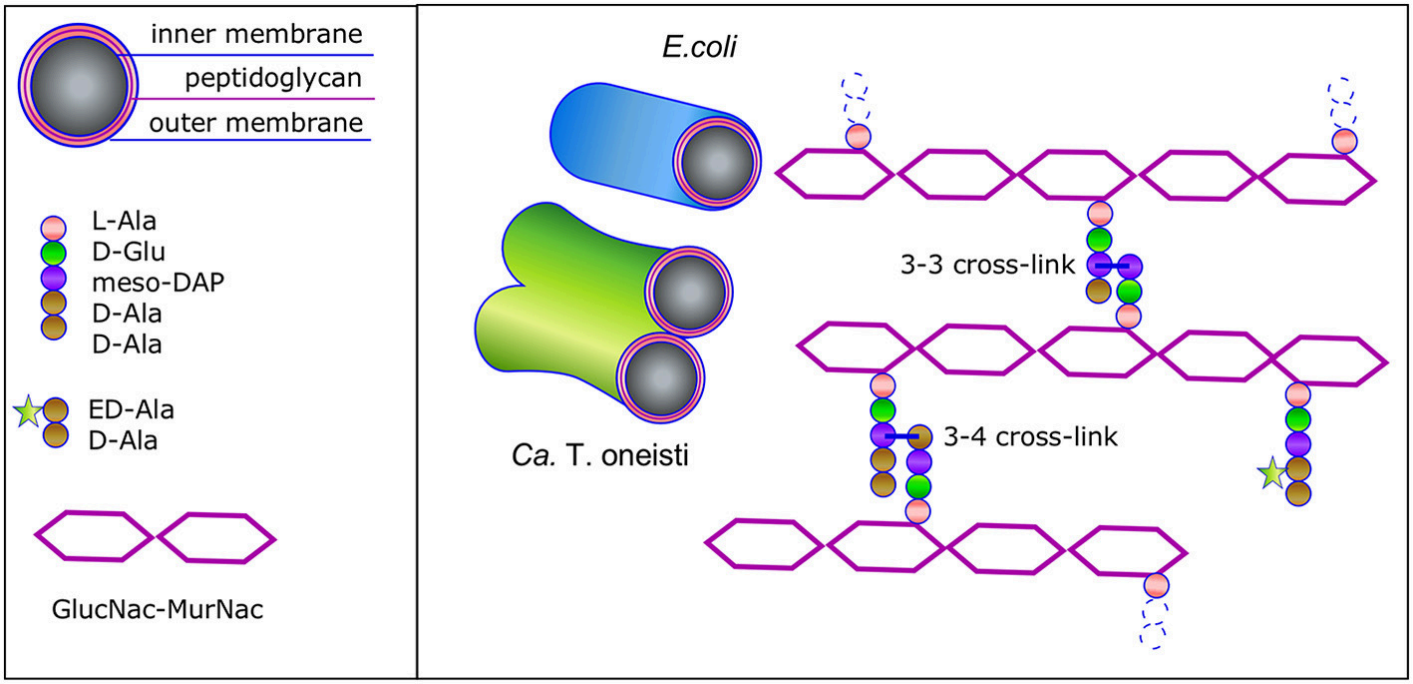
Schematic representation of the Gram-negative envelope and its peptidoglycan structure. Gram-negative bacteria have a three-layered envelope. The outer and inner membrane sandwich the single peptidoglycan (PG) layer. The glycan strands are connected by stem peptides that are either 3-4 or 3-3 crosslinked. ED-Ala-D-Ala (Liechti et al., 2014) is a clickable probe that is imported in the cytosol of the Thiosymbon and incorporated in the N-acetyl muramyl(MurNac)pentapeptide. Next the precursor is coupled to the lipid tail undecaprenyl phosphate by MraY and to N-acetyl glucosamine (GlcNac) by MurG to complete the synthesis of Lipid II. The latter is then flipped across the cytoplasmic membrane and the PG precursor is incorporated in the peptidoglycan layer. Consequently, the nascent peptidoglycan synthesis can be monitored by chemical addition of a fluorophore.

The peptidoglycan (PG) layer protects bacteria against osmotic and chemical stress and is an absolute requirement to maintain shape for bacteria that live in a hypo-osmotic environments such as the sea and the soil, in contrast to species such as *Chlamydia* that live in Eukaryotic cells, which cytosol is osmotically in balance with their own cytosol (Liechti et al., 2016). The PG layer consists of glycan strands of repeating *N*-acetylglucosamine and *N*-acetylmuramic acid disaccharides of which the latter are interconnected by peptide side bridges (Egan et al., 2015). The fourth and third amino acid of two stem peptides are crosslinked. Alternatively, some bacterial species such as *Mycobacteria* (Gupta et al., 2010) and *Clostridia* (Peltier et al., 2011) make predominantly crosslinks between the third amino acid on both stem peptides (Figure 2). The PG network is present as a single layer in Gram-negative bacteria that have an outer membrane and as multiple layers in Gram-positive bacteria that do not have an outer membrane. The well-known Penicillin Binding Proteins (PBP)s that can be inhibited by *beta*-lactams are responsible for the synthesis of peptidoglycan (Vollmer and Bertsche, 2008), together with the penicillin insensitive glycosyl transferases (GTases) RodA and possibly FtsW (Meeske et al., 2015, 2016; Cho et al., 2016). The PG disaccharide pentapeptide building units are synthesized in the cytoplasm and coupled to a lipid linker (undecaprenyl pyrophosphate) by the integral membrane protein MraY to become attached to the cytoplasmic membrane as lipid II, and subsequently flipped to the outer side of the cytoplasmic membrane to be used by the PG synthases (Egan et al., 2015). PG forms a covalently closed network, which intactness is essential for the survival of the cells. Therefore, a well-regulated balance between the insertion of new PG and the cleavage of bonds in the existing layer is needed to allow for the growth and survival of the bacteria. This balance is achieved by the formation of large protein complexes, termed elongasome and divisome for cell elongation and division, respectively (Blaauwen et al., 2008).

## FtsZ

Septum synthesis and cell division is initiated by a cytoskeletal protein, the tubulin homolog FtsZ. It polymerizes in a GTP dependent fashion to form protofilaments of about 100 nm in length underneath the cytoplasmic membrane at midcell. In *E. coli*, the polymers are attached to the membrane by the bitopic membrane protein ZipA (Hale and de Boer, 1997) and the membrane associated protein FtsA (Tormo and Vicente, 1984). The FtsZ filaments are connected to each other by ZapA and possibly by other Z-ring organizing proteins such as Zap C, D, and E (Blaauwen et al., 2017). This structure has been termed the proto-ring because initially FtsZ was thought to form a continuous ring based on low resolution fluorescence microscopy. High resolution microscopy such as PALM, STET and SIM has revealed that the proto-ring is discontinuous and that FtsZ polymers likely form a loosely organized network of filaments in three dimensions (Strauss et al., 2012; Buss et al., 2013, 2015; Holden et al., 2014; Jacq et al., 2015), which are dynamically kept together by the aforementioned proteins. The FtsZ polymers were shown to grow on one end and release monomers on the opposite end of the filament, causing them to treadmill (Loose and Mitchison, 2014; Bisson-Filho et al., 2017; Yang et al., 2017). After a measurable delay (Aarsman et al., 2005; Gamba et al., 2009; Wu et al., 2018), the proto-ring recruits all proteins that are needed to coordinate the precisely regulated synthesis and cleavage of the septum that will become the new pole of the two daughter cells. The treadmilling of the FtsZ filaments seems to move the PG synthetic complexes in a circumferential direction guiding the synthesis of the septum. Cephalexin-mediated Inhibition of transpeptidase activity, does not inhibit this movement (Yang et al., 2017). When the class B PBP3 responsible for the crosslinking of the glycan strands during cell division is specifically inhibited by aztreonam (Kocaoglu and Carlson, 2015) the cells are not able to divide, and form filaments while the division machinery remains localized at midcell for at least two mass doublings (Pogliano et al., 1997; Blaauwen et al., 2003; Potluri et al., 2010). Possibly the division machinery does not dissociate while PBP3 is inhibited, because glycosyl transferase (GTase) activity continues in a futile cycle of synthesis and hydrolysis of the glycan strands until the lipid II precursors are depleted (Cho et al., 2014). FtsZ mutants that have a reduced GTPase activity and therefore reduced depolymerization rate, slow down the movement of the PG synthases (Yang et al., 2017), suggesting that FtsZ treadmilling directs PG synthesis.

**Figure 3:**
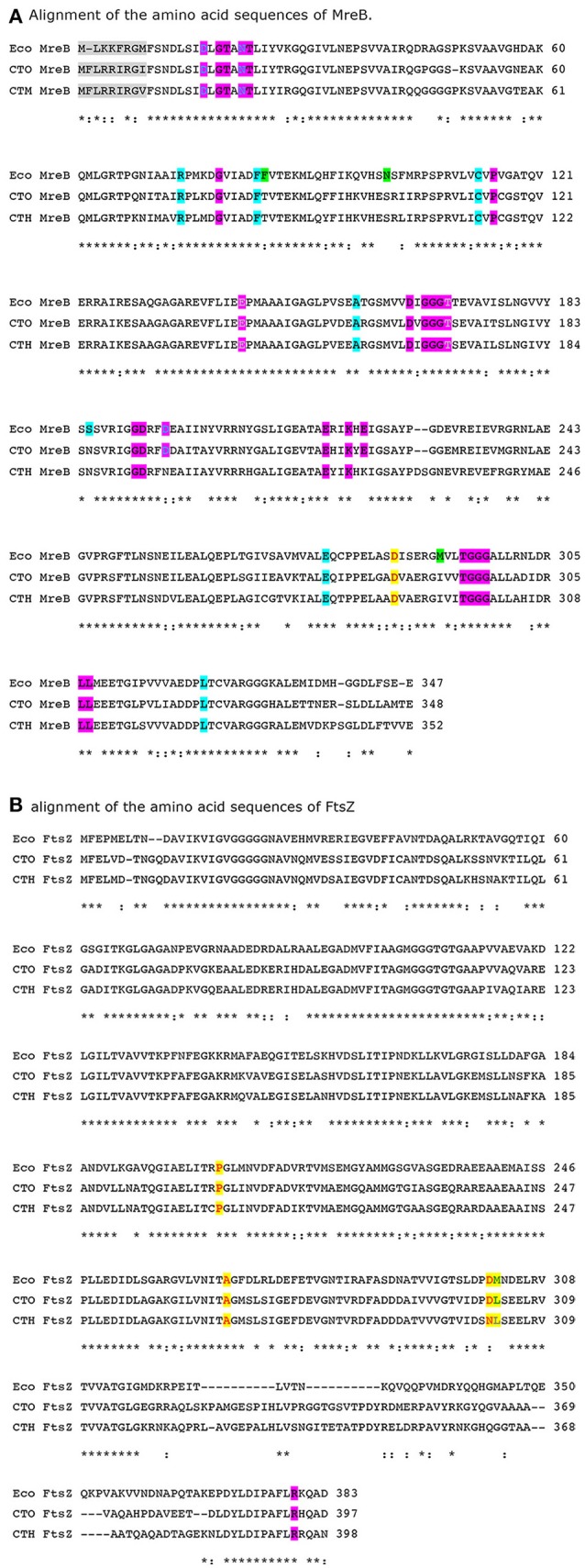
**(A,B)** Alignment of *E. coli* (Eco), *Ca*. T. oneisti (CTO) and *Ca. T. hypermnestra* (CTH) MreB and FtsZ. Active site residues in MreB are highlighted in magenta (van den Ent et al., 2001). Amino acids that are in direct contact with A22 are shown in gray (Bean et al., 2009). Amino acids that confer A22 resistance are highlighted in cyan or when overlapping with ATP binding shown in magenta (Gitai et al., 2005; Dye et al., 2011; Ouzounov et al., 2016). Amino acids in MreB that increase the width of *E. coli* are highlighted in green (Shi et al., 2017). Amino acids involved in the interaction of MreB with FtsZ are highlighted in yellow. The amino acid R379 that is essential for degradation of FtsZ by ClpXP in *E. coli* (highlighted magenta) is also conserved in the symbionts. Alignment was made by Clustal Omega (Sievers et al., 2011).

The Gram-negative gammaproteobacteria *E. coli, Ca*. T. oneisti, and *Ca*. T. hypermnestrae all have closely related FtsZ proteins (Figure 3) with 74% identical amino acid residues (amino acid residues 1-316, excluding the C-terminal variable region). The longitudinal mode of division of the symbionts is also supported by FtsZ, which forms an interrupted ellipse instead of a ring in *Ca*.T. oneisti (Leisch et al., 2012) and starts as an arc in the basal pole of *Ca*. T. hypermnestrae (Leisch et al., 2016; Figure 1A). The localization patterns of the Z-ring in these bacteria exemplify that a closed ring structure is not a requirement for division.

## MreB

In rod-shaped bacteria such as *E. coli* and *B. subtilis* two modes of PG synthesis can be discriminated. Elongation occurs by dispersed insertion of new material into the existing PG layer, and division is achieved by the synthesis of a division septum, which is later converted to yield two entirely new cell poles (De Pedro et al., 1997). The second cytoskeletal protein in most rod shaped bacterial species is MreB, an actin homolog that polymerizes as short filaments in an ATP dependent fashion, which attaches to the cytoplasmic membrane with its amphipathic helix (Salje et al., 2011). The distribution of MreB filaments over the entire length of the cell is even (Jones et al., 2001; Carballido-López and Errington, 2003). The filaments themselves are almost perpendicularly oriented to the width axis of the cell (Wang et al., 2012; Olshausen et al., 2013). It is not known what exactly determines this organization. In addition to its own interaction with the membrane, MreB is also attached by its interaction with the bitopic membrane protein RodZ (Shiomi et al., 2008), the integral protein MreD and the bitopic protein MreC that forms a similar organization as MreB in the periplasm (Leaver and Errington, 2005; van den Ent et al., 2006). The MreB filaments of about 300–500 nm move along the membrane with an average speed of half a cell circumference per min in *B. subtilis* and in *E. coli* (Domínguez-Escobar et al., 2011; Garner et al., 2011; van Teeffelen et al., 2011; Olshausen et al., 2013; Ouzounov et al., 2016). In contrast to the treadmilling of FtsZ, inhibition by mecillinam of the transpeptidase activity of the class B PBP2, responsible for the crosslinking of the glycan strands during elongation, stops this movement despite the ongoing futile cycle of GTase and turnover activity (van Teeffelen et al., 2011; Cho et al., 2016). Depletion of Lipid II or induction of the expression of a GTase defective RodA mutant also inhibits the MreB movement (Cho et al., 2016), indicating that MreB movement and PG insertion in the lateral cell wall are coupled. In contrast, depolymerization of MreB by its inhibitor A22 does only affect the number of filaments but not the speed of movement of the filaments that were still intact (van Teeffelen et al., 2011; Olshausen et al., 2013; Lee et al., 2014). The overall consensus is that MreB recruits and organizes the PG synthesis machinery needed for elongation and that insertion of new PG is responsible for the movement of MreB.

**Figure 4:**
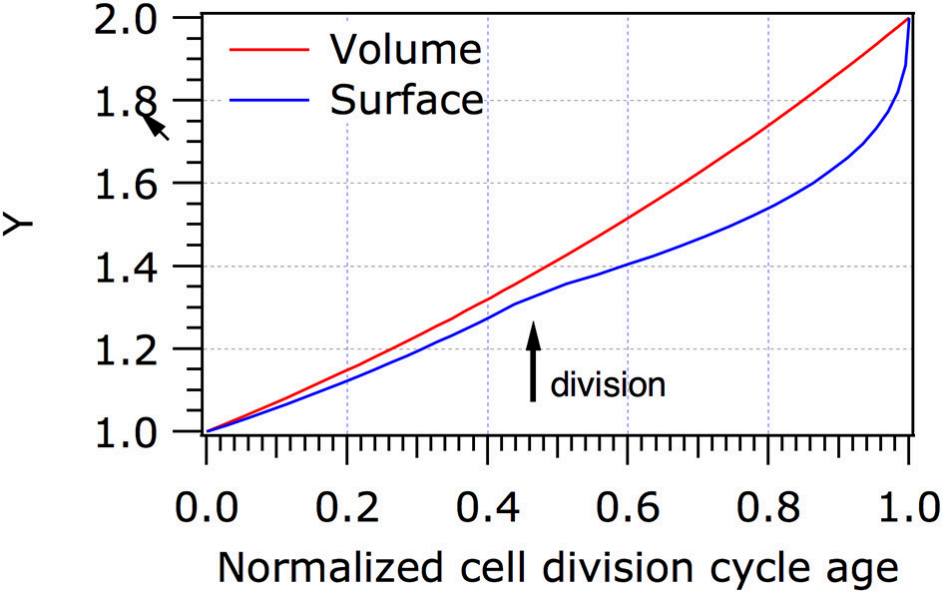
Increase in volume and surface of *Ca*.T. oneisti. When it is assumed that *Ca*. T. onesti is growing exponentially, it increases predominantly (70%) in surface during division. The normalized cell division cycle age is plotted against the volume (red) or the surface (blue) of the cells. Y represents the volume or the surface in arbitrary units. It should be noted that we have no information on the growth mode of the symbiont because they cannot be cultivated, apart from that they clearly grow in width only. The model is purely to show the putative contribution of division to the increase in width.

*Escherchia coli, Ca*. T. oneisti, and *Ca*. T. hypermnestrae all have closely related MreB proteins (Figure 3) with up to 77% identical and 88% similar residues. Surprisingly, the localization of MreB in the symbiotic bacteria is quite different from the well-known patchy distribution observed in *E. coli, B. subtilis* and *C. crescentus*. In non-dividing symbiont cells MreB was found in a very thin and restricted area in medially (parallel to the cell long axis) arranged patches (Pende et al., 2018). In dividing cells MreB seemed to associate initially with the Z-ellipse and both proteins co-localize. Upon invagination of the envelope, MreB and FtsZ separate in the proximal and distal poles where MreB reassumes its medial localization and FtsZ follows the constricting septum forming a ring and finally a dot in the almost separated daughter cells (Pende et al., 2018). Because of the large surface of the septum (Figure 1) in these longitudinally dividing bacteria, the doubling in volume assuming exponential growth comes to a large extent from the added surface area during cells division (see Figure 4 and Supplementary Video 1).

## The role of MreB in cell division

MreB filaments also run perpendicular to the long axis of *C. crescentus* cells, like in *E. coli*. In addition, MreB accumulates at midcell in dividing cells in an FtsZ dependent fashion (Figge et al., 2004; Gitai et al., 2004). In bacterial two hybrid experiments FtsZ was found to interact with MreB (White et al., 2010). Upon depletion of MreB, cell division was impaired and diameter control at midcell seemed to be lost implying that MreB is needed for cell division in *C. crescentus*. However, MreB leaves the cell division site at about the same time as the late localizing proteins such as FtsL and FtsW arrive (Goley et al., 2011), suggesting that MreB is only needed at an early stage in which the cells are preparing for cell division. A similar temporal midcell localization of MreB was observed in *E. coli* (Vats and Rothfield, 2007; van der Ploeg et al., 2013), and a specific interaction between FtsZ and MreB was shown *in vivo* and *in vitro* (Fenton and Gerdes, 2013). It was hypothesized that midcell localization of MreB might help to position the lipid II synthesizing complex at midcell because MurG, the last protein involved in lipid II synthesis, could be immunoprecipitated together with MreB in *E. coli* (Mohammadi et al., 2007; van der Ploeg et al., 2013). In *Caulobacter*, MurG and MreB are both dependent on the presence of a FtsZ ring for midcell localization and treatment of the cells with A22, which inhibits the polymerization of MreB does not affect the localization of MurG (Aaron et al., 2007). MurG midcell localization is not dependent on active PG synthesis in *Caulobacter* (Aaron et al., 2007). These data suggest that if MreB helps to recruit the lipid II synthesis complex to midcell its role is only temporarily.

Cells that do not possess FtsZ, such as *Chlamydia trachomatis*, require MreB to synthesize a temporal thin band of PG at the division plane (Liechti et al., 2016). The deltaproteobacterium *Bdellovibrio bacteriovorus* is a very small Gram-negative bacterial species that preys on Gram-negative species like *E. coli* (Kuru et al., 2017). It has two MreB proteins, one involved in elongation and one involved in division (Fenton et al., 2010). Interestingly, bacteria that grow by dispersed insertion of PG, do not always use MreB for this purpose. For instance, in *Rhodobacter sphaeroides* MreB localizes predominantly at a predivisional stage at the septum in elongating cells, and moves to future division sites when septation starts (Slovak et al., 2005). In the symbiont cells *Ca*. T. oneisti and *Ca*. T. hypermnestrae the MreB inhibitor A22 completely abolished the synthesis of peptidoglycan as monitored by the lack of incorporation of fluorescent PG precursors (Figure 2; Pende et al., 2018). In addition, the specific localization and assemblage of FtsZ is lost (Pende et al., 2018). Inhibition by A22 of MreB in *E. coli* results in spherical cells that increase in diameter (Karczmarek et al., 2007; Ouzounov et al., 2016). No change in the width of the symbiont cells was observed after prolonged incubation with A22. Since all PG synthesis stops under these conditions, also no shape changes are observed in the symbionts.

The concentration of FtsZ in non-dividing *Ca*. T. oneisti is only 10–20% of that in dividing cells (Leisch et al., 2012), which could indicate that it is specifically synthesized at the moment the cells are ready to divide. Amino acid residue R379, involved in degradation of FtsZ by the proteolytic system ClpXP in *E. coli* (Viola et al., 2017) is also conserved in the symbionts (Figure 3). Possibly a large part of FtsZ is degraded after the symbionts have finished division. As MreB is known to interact with FtsZ in *E. coli* and the involved amino acids in MreB as well as in FtsZ are conserved in *Ca*. T. oneisti, it is conceivable that MreB recruits FtsZ to the potential division site. Because these symbionts double in width predominantly by dividing (Pende et al., 2018), MreB might synthesize a band of preseptal PG and recruit FtsZ. Inhibition of MreB by A22 would not provide the band of preseptal peptidoglycan nor recruit FtsZ and perhaps fails to activate FtsZ synthesis. Consequently, all new peptidoglycan synthesis is completely abrogated by inhibiting MreB. The examples presented above suggest that MreB is directing specific PG synthesis at midcell in at least many Gram-negative bacterial species. The activity of MreB at midcell likely contributes to the maintenance of the correct diameter of the cells and to the precise positioning of their new division plane.

In *C*. T. oneisti FtsZ forms an ellipse that initially colocalizes with MreB. When constriction progresses, FtsZ remains concentrated at the midcell where new PG is synthesized, whereas the MreB signal splits in two, occupying the medial and future division sites of both daughter cells (Pende et al., 2018). PG synthesis during constriction requires a highly active synthesizing machinery to produce the new cell pole, which comprises 22% of the surface of new-born *E. coli* cells. In the symbionts, septal synthesis can surmount to an estimated 45–70% of the surface of the newborn cell (Figure 4 and Supplementary Video 1). Consequently, regions of active PG synthesis were predominantly observed at the leading edge of the constriction where FtsZ was localized (Pende et al., 2018).

## What's in a pole?

Conceivably, the symbiotic bacterial species have reoriented their PG synthesizing machinery such that the Z-ring is positioned parallel to the long axis of the cell, which would then correspond to the cylindrical part of an *E. coli* cell (Figure 1B model 2). The position where constriction is initiated would then be the equivalent of the cell poles in *E. coli*. This would require the reorientation of the major cytoskeletal elements MreB and FtsZ in the symbiont cells. An alternative interpretation could be that the symbionts are just very fat short *E. coli* cells (Figure 1B model 1). This would not require any reorganization of MreB and FtsZ. The medial position of MreB in the symbionts would then correspond to the cylindrical part of *E. coli*. Recently a study has been described in which MreB was randomly mutagenized and cells were selected based on their morphology by FACS (Shi et al., 2017). The mutations in MreB that resulted in very fat *E. coli* cells (almost 2 μm in width) are similar in the symbionts. For instance, the two mutations that cause the largest increase in width in *E. coli* M291V and F86S are an isoleucine (similar to valine) and a threonine (similar to serine) in *Ca*. T. oneisti as well as in *Ca*. T. hypermnestra. This illustrates that subtle changes might be sufficient to change at least one dimension of the morphology of the cells.

Since the symbionts in the latter interpretation would consist of almost only cell pole, the medial part in which MreB is localizing in a thin band would have the same orientation as the cylindrical part in *E. coli*. Since there is very little PG to be synthesized during elongation of the symbionts, MreB has obtained predominantly the function of marking the midcell position by the synthesis of a band of preseptal PG. Not excluding that it might contribute to the synthesis of the new cell poles during septation. FtsZ, is doing exactly what it is doing in *E. coli*, only it occupies a much larger circumference. By now it is clear that this is not really an issue as the Z-ring is discontinuous in all thus far investigated bacterial species (Blaauwen et al., 2017).

The cell poles in *E. coli* are thought to be inert with respect to PG synthesis (De Pedro et al., 1997) and so could be the very large putative poles of the symbionts. The observation in *E. coli* is based on the incorporation of gold labeled D-cysteine, which was diluted in density in the cylindrical wall but not in the cell poles during a chase for two mass doublings (De Pedro et al., 1997). Indicating that these D-cysteines were not accessible to PG turnover enzymes. Although many proteins involved in length growth are not present in the cell poles, many specialized sensor proteins are (for instance, the serine chemoreceptor, the DcuS/DcuR two-component system, the chemoreceptor Tar, and the Osmosensing transporter ProP; Scheu et al., 2014; Neeli-Venkata et al., 2016; Romantsov et al., 2017; Saaki et al., 2018). Thus, although poles of *E. coli* are inert for PG recycling, they function fully in perception of their environment. *Ca*. T. oneisti and *Ca*. T. hypermnestra cannot be cultivated, which makes experiments with living cells very limited. Consequently, at this moment it is not known whether the very large putative poles of the symbionts are inert with respect to PG turnover. Even if they were, proteins required for nutrient uptake and environmental sensing could be inserted during new cell pole synthesis. In addition, it is not known whether limited reorganization of polar PG occurs in *E. coli*. For instance, for the insertion of envelope spanning proteins. The wealth of available PG hydrolases in *E. coli* (Vollmer et al., 2008) and in the symbionts, could allow the bacteria to make new pores in the PG layer by hydrolysis of the 3–4 crosslinks and insert new protein complexes or remove proteins complexes and subsequently repair the PG layer by creating 3–3 crosslinks (Figure 2; Hugonnet et al., 2016; Kuru et al., 2017). This provides sufficient flexibility to insert whatever is needed in the envelope of the symbionts. This leaves the very short length axis of the symbionts of which the top and bottom look like a pole as the only available place for nascent PG synthesis, which is exactly what is observed in the symbiont cells (Pende et al., 2018).

MreB is reported to avoid the poles of *E. coli* because of the presence of anionic phospholipids such as cardiolipin and phosphatidylglycerol, which are there because of the increased curvature of the poles (Renner et al., 2013; Billings et al., 2014; Ursell et al., 2014; Kawazura et al., 2017). However, in a case where MreB was forced to bind to the poles it would stimulate PG synthesis and create Y-shaped cells, which is how division starts in *Ca* T. hypermnestra (Kawazura et al., 2017). Presently it is unknown what the lipid composition is of symbiont envelopes, but marine gammaproteobacteria, especially sulfuroxidizing bacteria, can have a very different lipid composition (Sebastián et al., 2016; Favre et al., 2018). For MreB to localize in the top or bottom of the symbiont cell, where the curvature is high, possibly one of the requirements is to adapt the lipid composition. The amount of RodZ is reported to affect the sensitivity of MreB for curvature (Shi et al., 2018). The organization and amount of RodZ and MreCD (only ± 15% amino acid identity with *E. coli* versions) in the symbionts might be able to overrule the negative influence of the membrane curvature for MreB localization. For the symbionts to survive on the cuticle of the host longitudinal division was an essential adaptation. In general, evolution works best if increased fitness can be achieved by relatively small changes. MreB and FtsZ of the symbionts are very conserved and their minor changes might explain some of the changes in morphology, but likely the adaptation are to be found in the other proteins that are part of the elongasome and divisome. The alternative model (Figure 1B model 2) in which the symbionts have swapped their axes and the orientation of their cytoskeletal proteins would require different adaptations. For instance, what would prevent MreB to orient perpendicular to their short axis and what would restrict MreB to the medial position only? During septation, the synthetic machinery should discriminate between the top and bottom of the cells, which would have to become inactive with respect to PG synthesis and the envelope in between these parts should remain active areas of PG synthesis. Due to our present lack of knowledge on the morphogenesis of the symbionts, it is not possible to choose between the two models. However, from an evolutionary point of view, Model 1 in Figure 1B might be realized with the least complicated adaptations.

## Author contributions

The author confirms being the sole contributor of this work and approved it for publication.

### Conflict of interest statement

The author declares that the research was conducted in the absence of any commercial or financial relationships that could be construed as a potential conflict of interest.

## Supplementary material

Supplementary Video 1 **Simulation of the growth and division of a *Ca*. Thiosymbion oneisti cell**.
